# Supplementary material for: Cross-platform transcriptional profiling identifies common and distinct molecular pathologies in Lewy body diseases
Source: Acta Neuropathol. 2021 Jul 26;142(3):449–74. doi: 10.1007/s00401-021-02343-x (PMC8357687; doi:10.1007/s00401-021-02343-x)
Supplement: Supplementary file 1 — Supplementary file1 (DOCX 3224 KB) [file 401_2021_2343_MOESM1_ESM.docx]

# Supplementary Figures


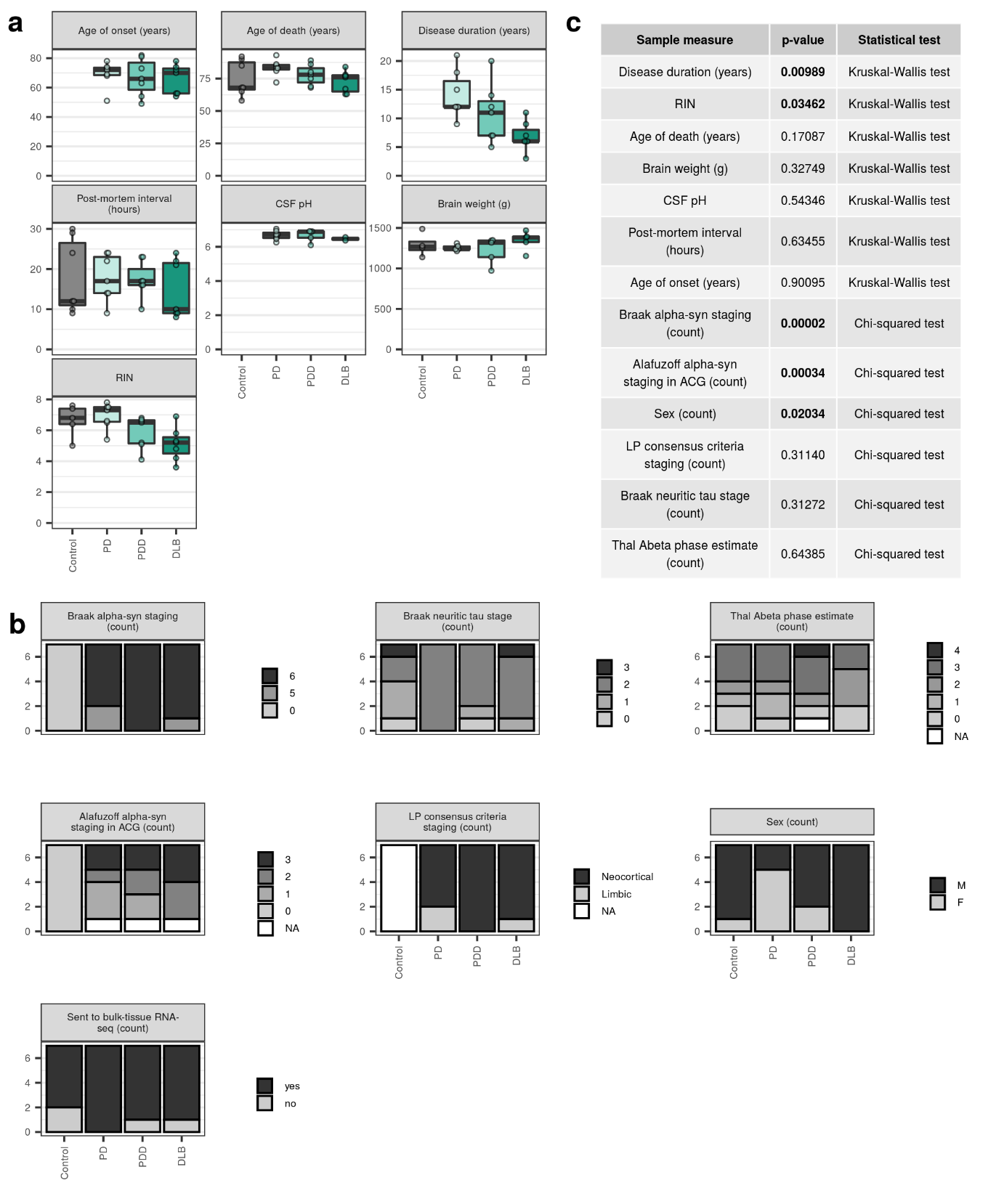


Supplementary Fig. 1 Subject demographics, sample information and pathological measures.

Continuous (a) and categorical (b) subject demographics, sample measures and pathological measures are shown for each disease group. (c) Significant differences in groups were tested using either the Kruskal-Wallis rank sum test (for continuous variables) or the Chi-squared test (for categorical variables). Significant differences (p < 0.05) are shown in a bold face. All measures per individual are available in Supplementary Table 1. Abeta, amyloid β; ACG, anterior cingulate gyrus; alpha-syn, alpha-synuclein; CSF, cerebrospinal fluid; LP, Lewy pathology; RIN, RNA integrity number.


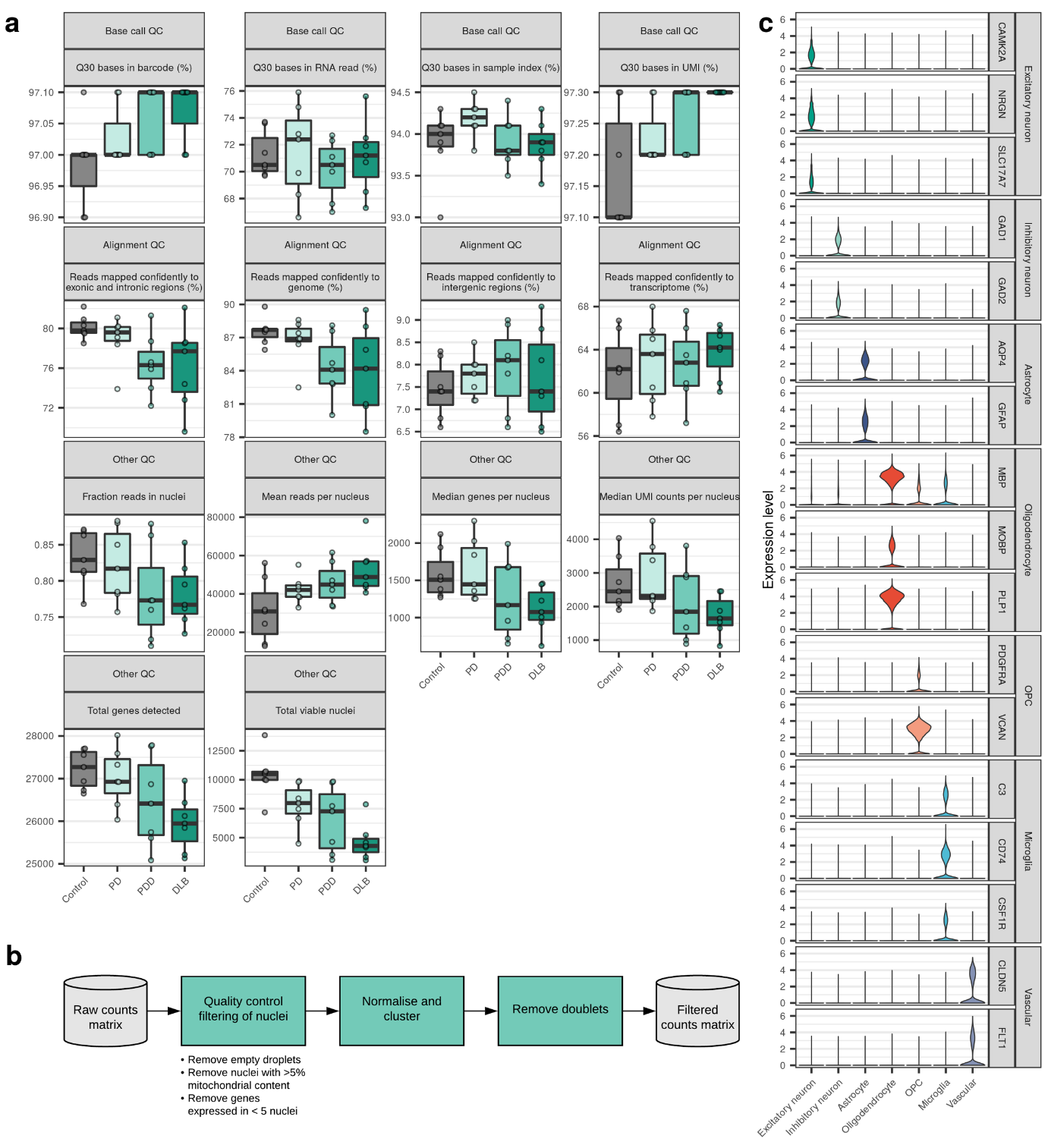


Supplementary Fig. 2 Single-nucleus RNA-sequencing metrics, quality control and cell-type classification. (a) Single-nucleus sequencing metrics from Cell Ranger, grouped by the type of quality control (QC) metric they represent i.e. base call, alignment or other. All metrics per sample are available in Supplementary Table 1. (b) Workflow illustrating the steps taken to filter the raw data to only include true nuclei of high quality. These steps were independently applied across each of the 28 sequenced samples. (c) Violin plots of expression values (y-axis) for known cell-type marker genes in each of the cell-type clusters identified (x-axis). OPC, oligodendrocyte precursor cell; UMI, unique molecular identifier.


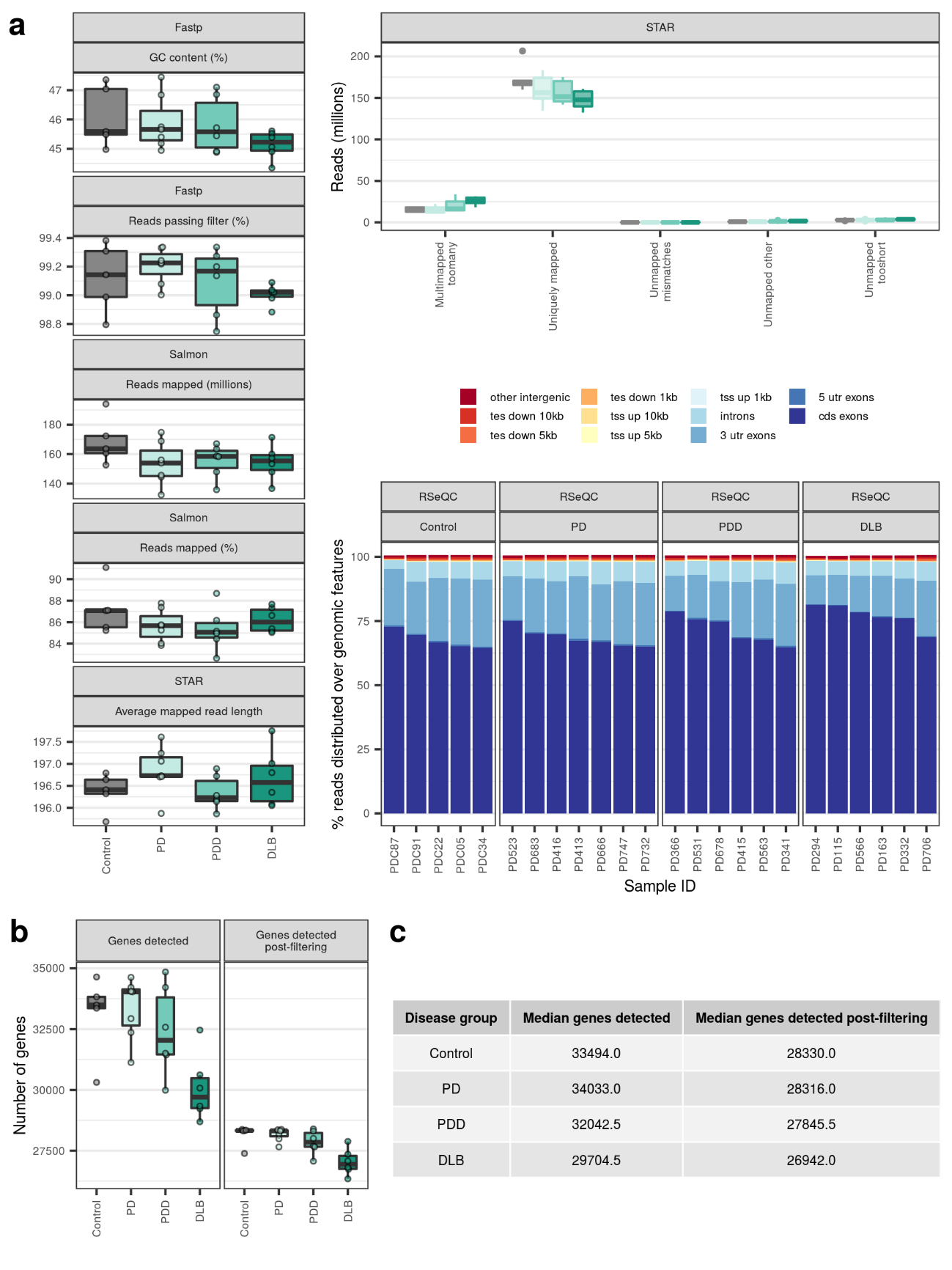


Supplementary Fig. 3 Bulk-tissue RNA-sequencing metrics.

(a) Bulk-tissue RNA-sequencing metrics from Fastp, STAR, Salmon and RSeQC. (b) Number of genes detected in bulk-tissue RNA-sequencing of each sample before and after filtering. Detection of a gene before filtering was defined as a count > 0 in at least one sample across a disease group, while detection of a gene after filtering was defined as a count > 0 in all samples across a disease group. Only genes that were detected after filtering were used for bulk RNA-sequencing differential gene expression analyses. (c) Descriptive statistics for (b). All metrics per sample are available in Supplementary Table 1. CDS, coding sequence; GC, guanine-cytosine; TES, transcription end site; TSS, transcription start site; UTR, untranslated region.


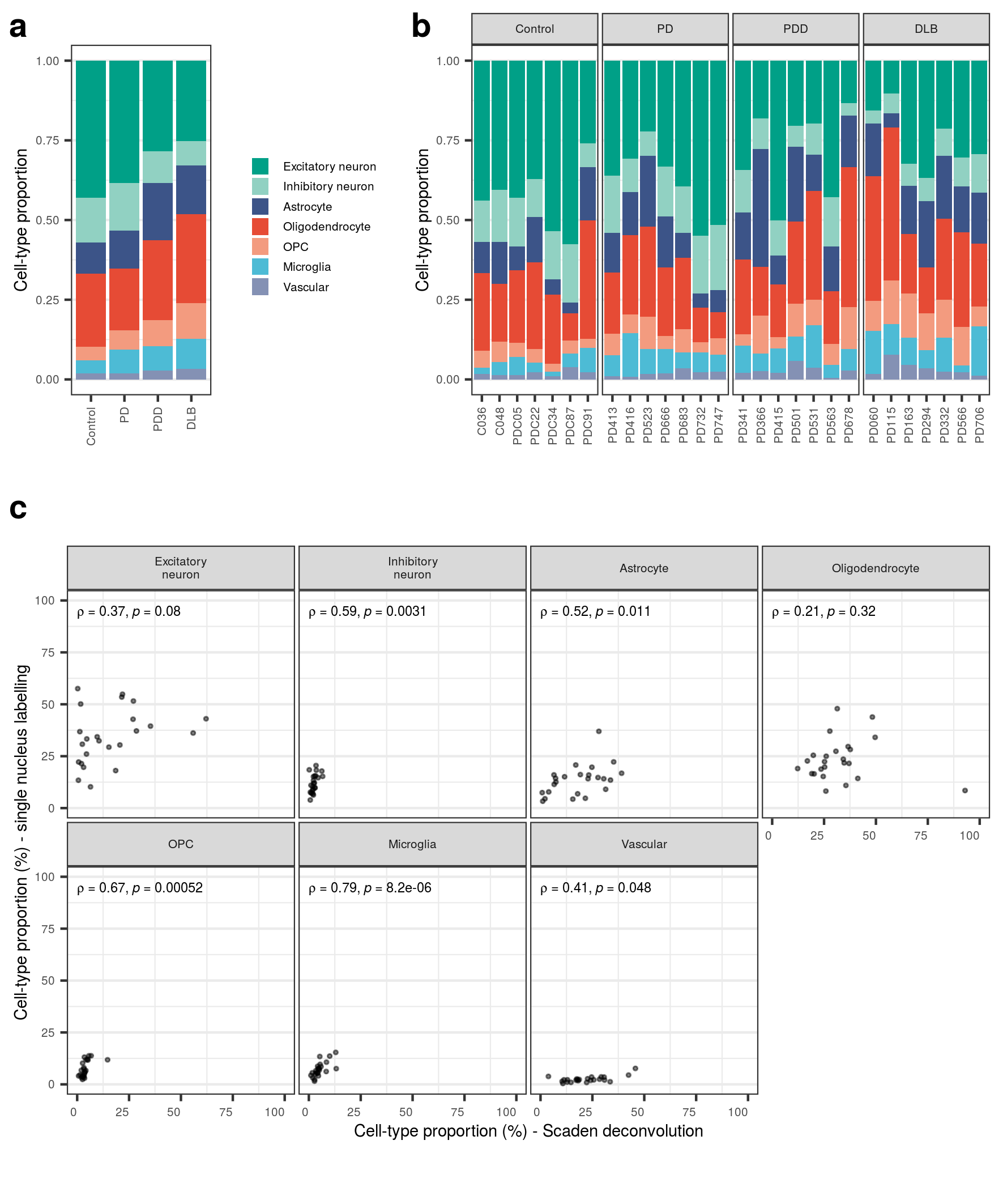


Supplementary Fig. 4 Consistency of cell types across individuals.

(a) Proportion of nuclei of each cell type isolated across all individuals in each disease group (n = 7 per group) and (b) across each individual. (c) Scatterplot of cell-type proportions (%) derived from Scaden deconvolution and cell-type labelling of single nuclei. In each panel, Spearman’s rho (ρ) and associated p-value (p) are displayed. Proportions are available in Supplementary Table 2. OPC, oligodendrocyte precursor cell.


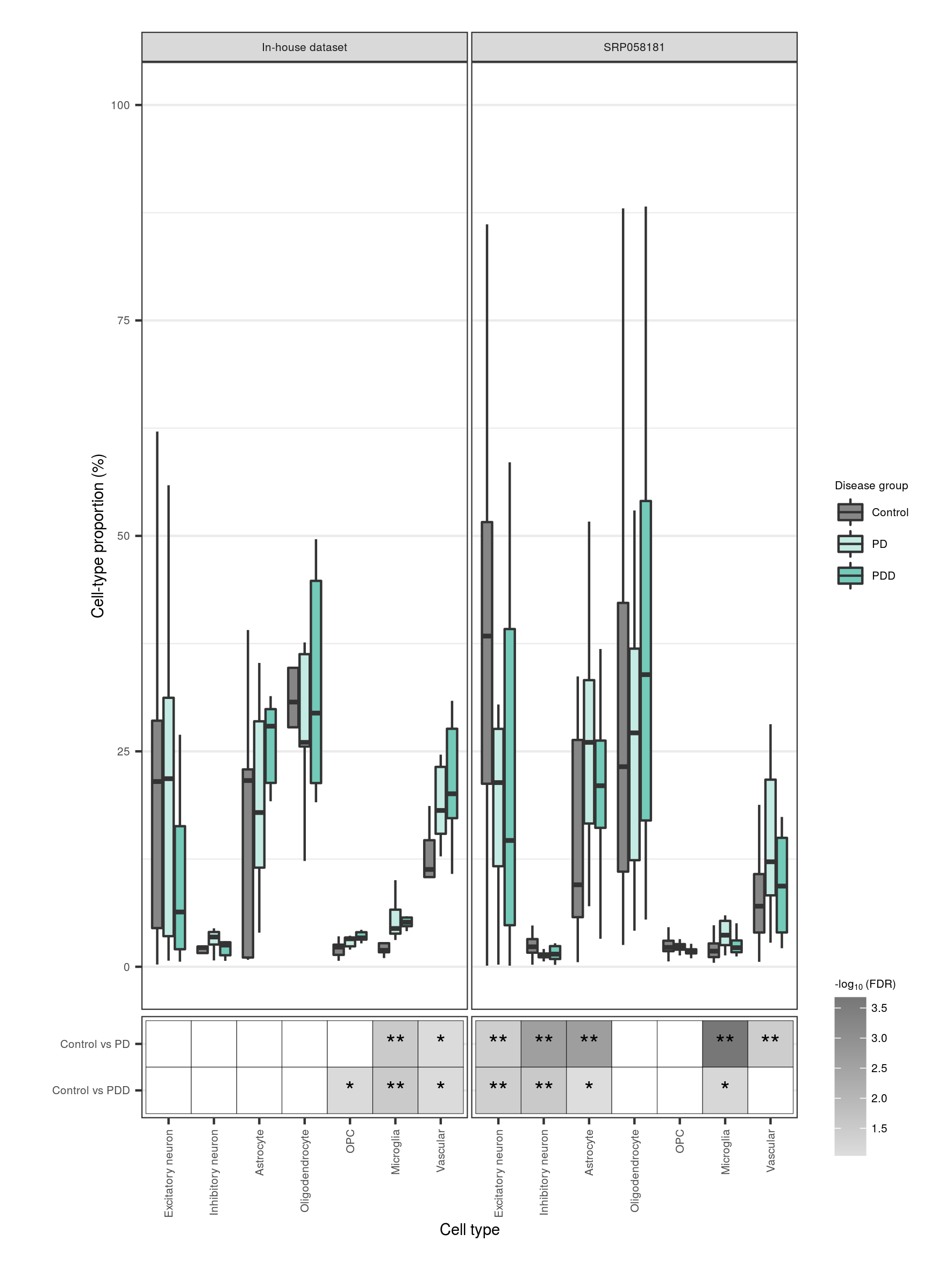


Supplementary Fig. 5 Replication of cell-type proportions.

Cell-type proportions derived from Scaden deconvolution of our bulk-tissue RNA-sequencing or the recount dataset, SRP058181. Cell-type proportions (upper panel) are grouped by cell type and disease status and displayed relative to the median of controls (within a cell type). Significant differences in cell-type proportions between disease groups (lower panel) were determined using the Wilcoxon rank sum test, with FDR correction for multiple testing. Non-significant results (FDR > 0.1) were coloured white; **, FDR < 0.05; *, FDR <= 0.1. Results are available in Supplementary Table 2. OPC, oligodendrocyte precursor cell.


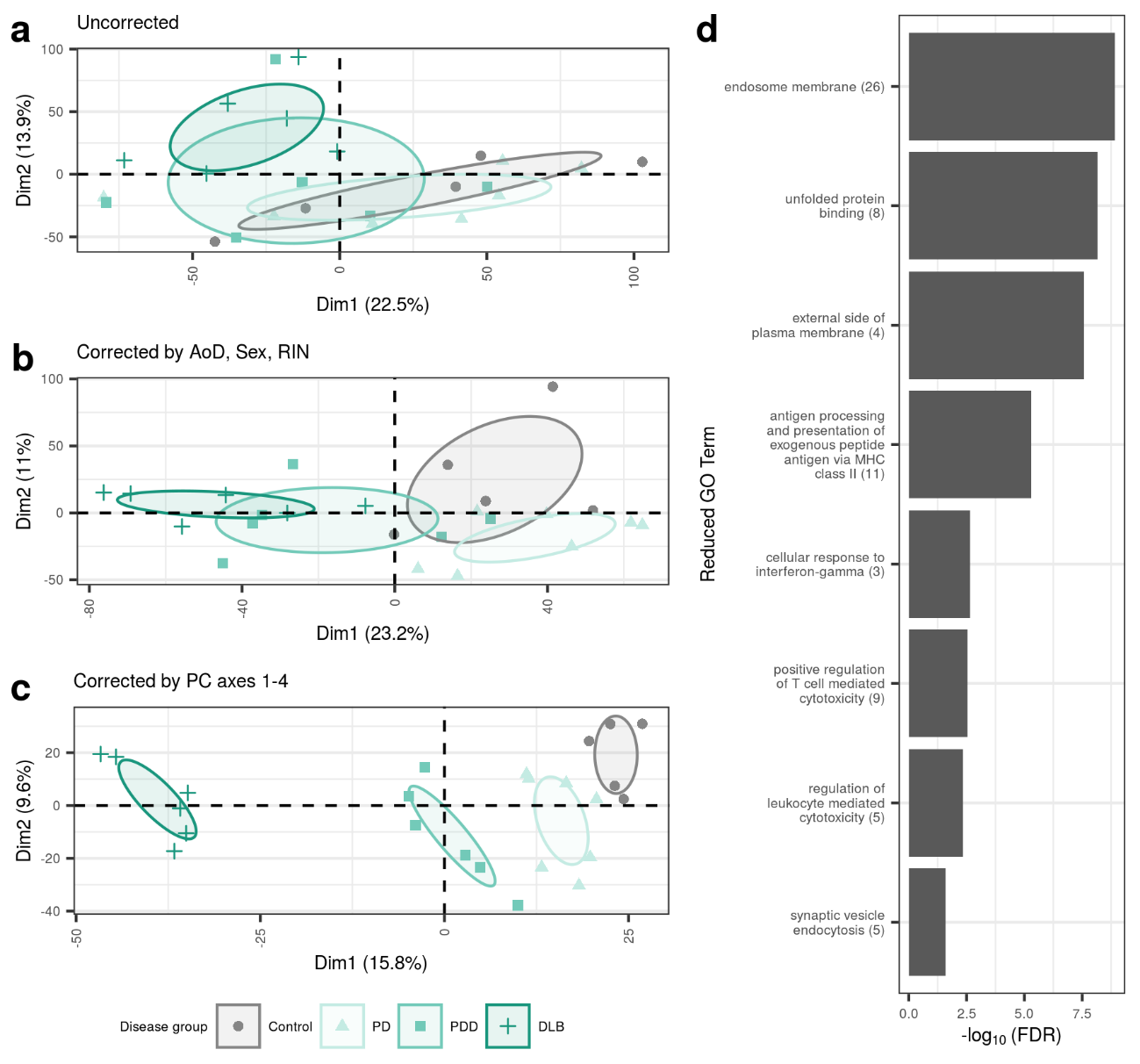


Supplementary Fig. 6 Effect of different batch-correction strategies on sample clustering by gene expression.

Samples were plotted by the first two principal components (otherwise known as dimensions, dim) derived from (a) uncorrected gene expression, (b) gene expression adjusted for age of death, sex and RIN and (c) gene expression adjusted for cell-type and experimental covariates. Principal component analyses were performed on gene-level expression filtered to include only genes with count > 0 in all samples (28,692 genes). Ellipses represent the 95% confidence level around group mean points (not displayed). (d) Reduced gene ontology (GO) terms associated with the top 100 genes that contribute to the first principal component (PC1) in (c). Original GO term enrichments (referred to as “child terms”) were grouped using semantic similarity. The number of enriched child GO terms assigned to each parent term is indicated in parentheses on the y-axis. Each parent term is represented by the most significant child term associated with it. Top 100 genes, their contributions to PC1 and results of pathway enrichment are available in Supplementary Table 4.


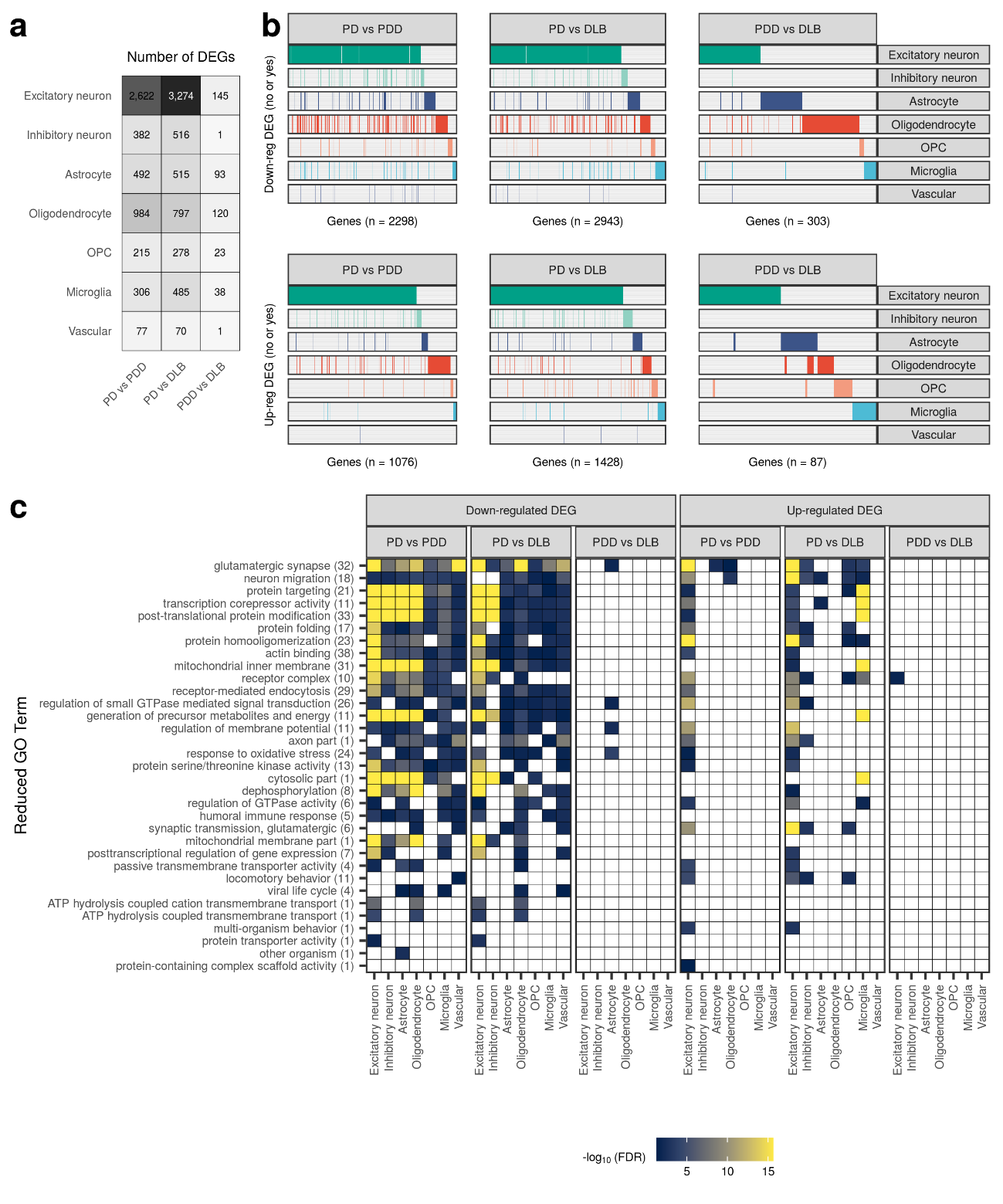


Supplementary Fig. 7 Cell-type-specific gene expression changes and pathway enrichments between disease states.

(a) Number of differentially expressed (DE) genes (|log_2_(fold change)| ≥ log_2_(1.5), FDR < 0.05) across each cell type in pairwise comparisons between disease groups. The fill of the square is proportional to the number of DE genes. (b) Binary plot indicating with bars whether a gene (column) is down-regulated (upper panel) or up-regulated (lower panel) in a given cell type (rows). Number of DE genes in each comparison indicated on the x-axis. (c) Reduced GO terms associated with cell-type-specific down- and up-regulated DE genes identified across pairwise comparisons between disease groups. Due to the magnitude of pathway enrichments, original GO term enrichments (referred to as “child terms”) were grouped using semantic similarity. The number of enriched child GO terms assigned to each reduced parent term across all cell types and comparisons in the panel is indicated in parentheses on the y-axis. Reduced GO terms were ordered on the y-axis by the number of cell types and comparisons in which the term was found enriched. The fill of each tile indicates the -log_10_(FDR) of the most significant child term associated with the parent term within that comparison/cell type. Non-significant results (FDR > 0.05) were coloured white. All cell-type-specific DE genes and pathway enrichments are available in Supplementary Table 5 and Supplementary Table 6, respectively. DEG, differentially expressed gene; GO, gene ontology; OPC, oligodendrocyte precursor cell.


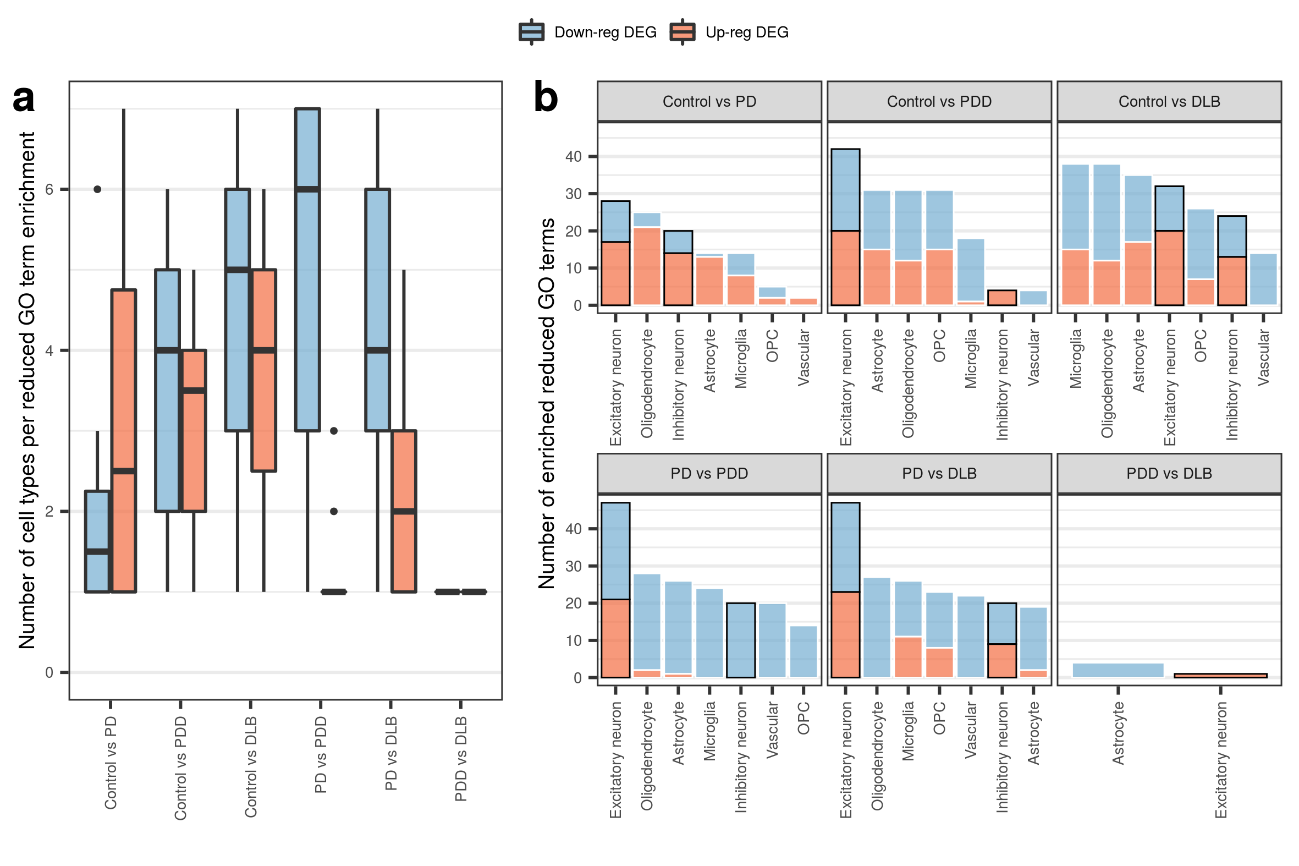


Supplementary Fig. 8 Reduced GO term counts across cell types and pairwise comparisons.

(a) Number of cell types each gene ontology (GO) term was associated with using cell-type-specific down- and up-regulated DE genes identified across pairwise comparisons. (b) Number of enriched GO terms identified using cell-type-specific down- and up-regulated DE genes from each pairwise comparison. Within each panel, cell types are ordered by the total number of enriched GO terms, irrespective of direction of effect, from highest to lowest. Excitatory and inhibitory neurons have been marked with a black border. DEG, differentially expressed gene; OPC, oligodendrocyte precursor cell.


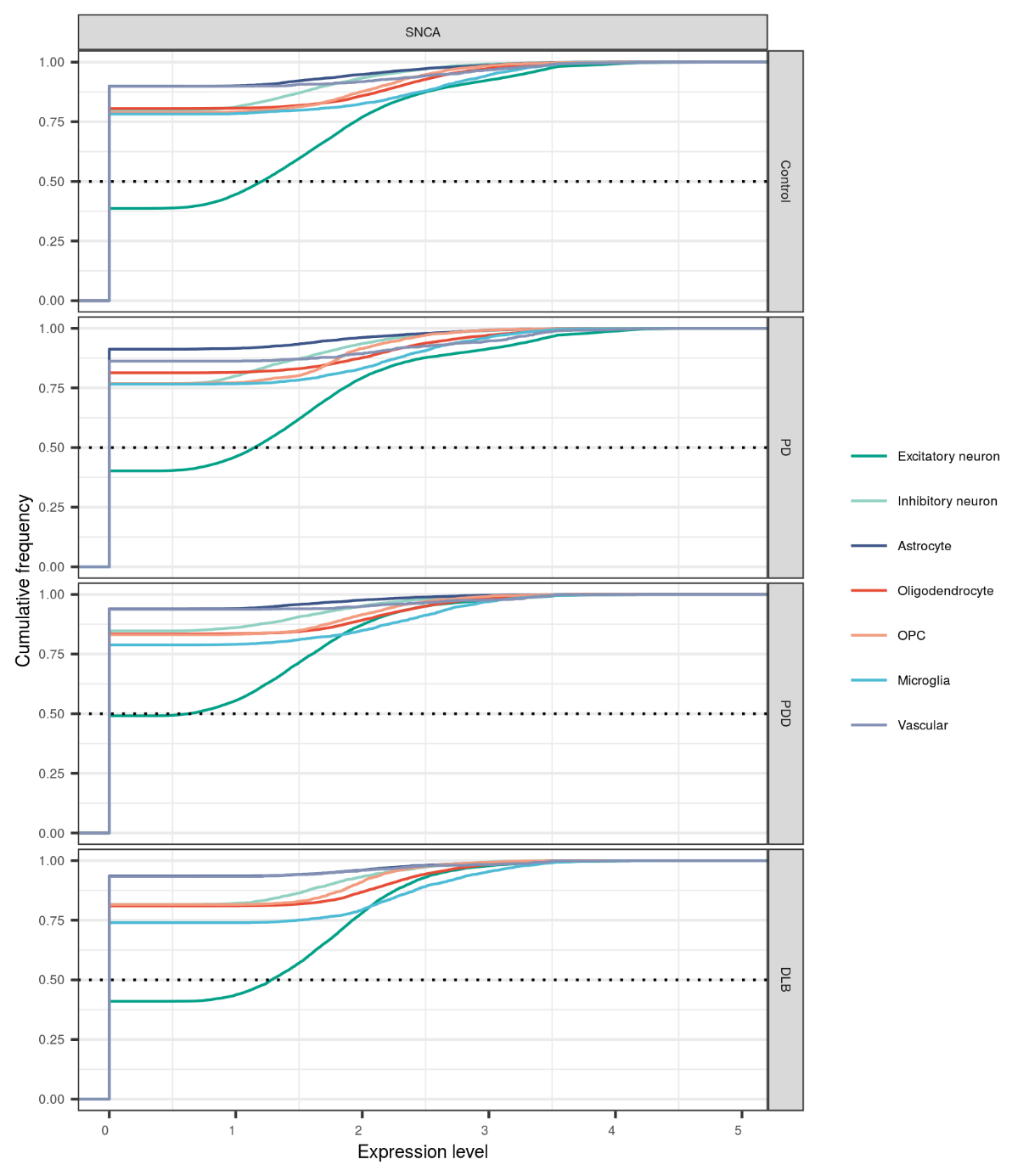


Supplementary Fig. 9 *SNCA* expression across cell types in each disease group.

Cumulative distribution plot comparing *SNCA* expression levels in each disease group across cell types. Cumulative distribution plots display the proportion of data (y-axis) less than or equal to a specified value (x-axis). The horizontal dashed line denotes where 50% of the data lies. OPC, oligodendrocyte precursor cell.


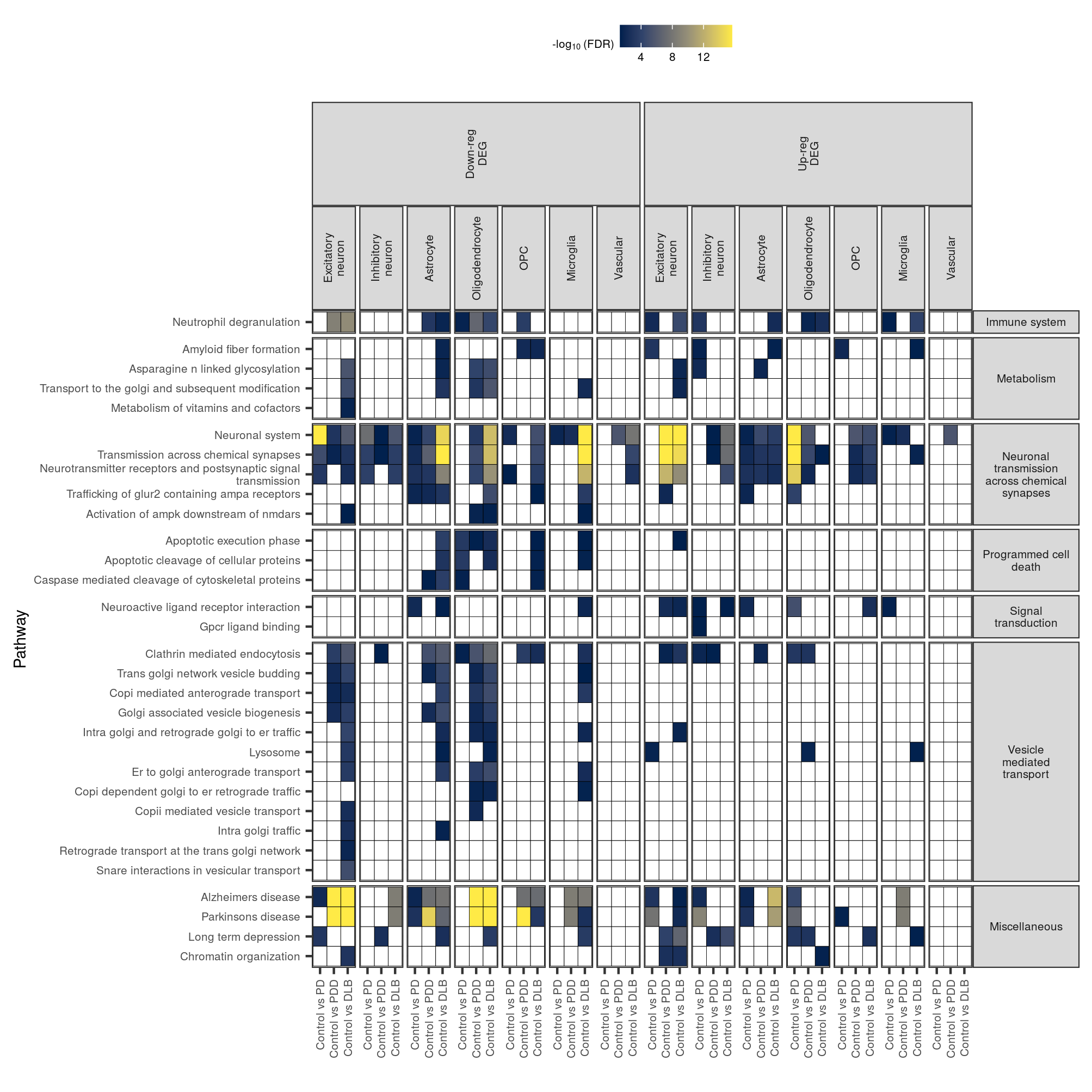


Supplementary Fig. 10 Cell-type-specific pathway enrichments across pathways genetically associated with PD.

Pathway enrichments for all 46 PD-associated pathways (associated in a large-scale polygenic risk score-based assessment of 2,199 gene sets). The fill of each tile indicates the -log_10_(FDR) of enrichment. Non-significant results (FDR > 0.05) were coloured white. Pathway enrichment results are available in Supplementary Table 7. DEG, differentially expressed gene; OPC, oligodendrocyte precursor cell. PD-associated pathways were derived reference [48].


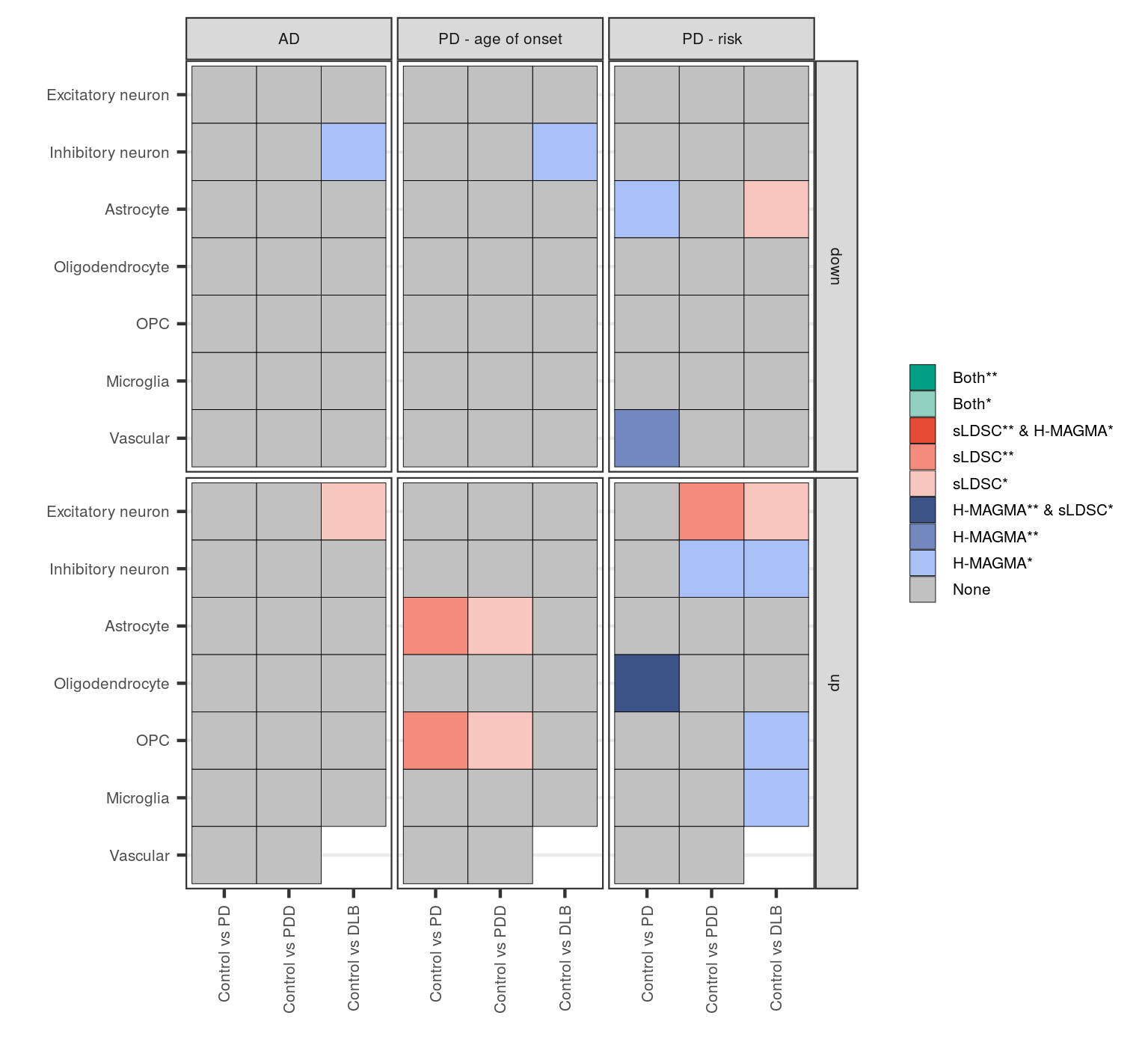


Supplementary Fig. 11 Genetic associations with cell-type-specific differentially expressed genes split by direction of effect.

Genetic associations using down- and up-regulated cell-type-specific differentially expressed genes in disease comparisons with controls. Two methods were used to identify associations: Hi-C-coupled MAGMA (MAGMA) and stratified LD score regression (sLDSC). The heatmap is coloured by degree of significance with both or either method, with * and ** indicating nominal significance (unadjusted p-value < 0.05) or significance (FDR-corrected p-value < 0.05; corrected for number of cell types tested). Only 2 genes were up-regulated in vascular cells from Control vs DLB, thus no results were returned from either analysis. Results available in Supplementary Table 8. AD, Alzheimer’s disease; OPC, oligodendrocyte precursor cell.


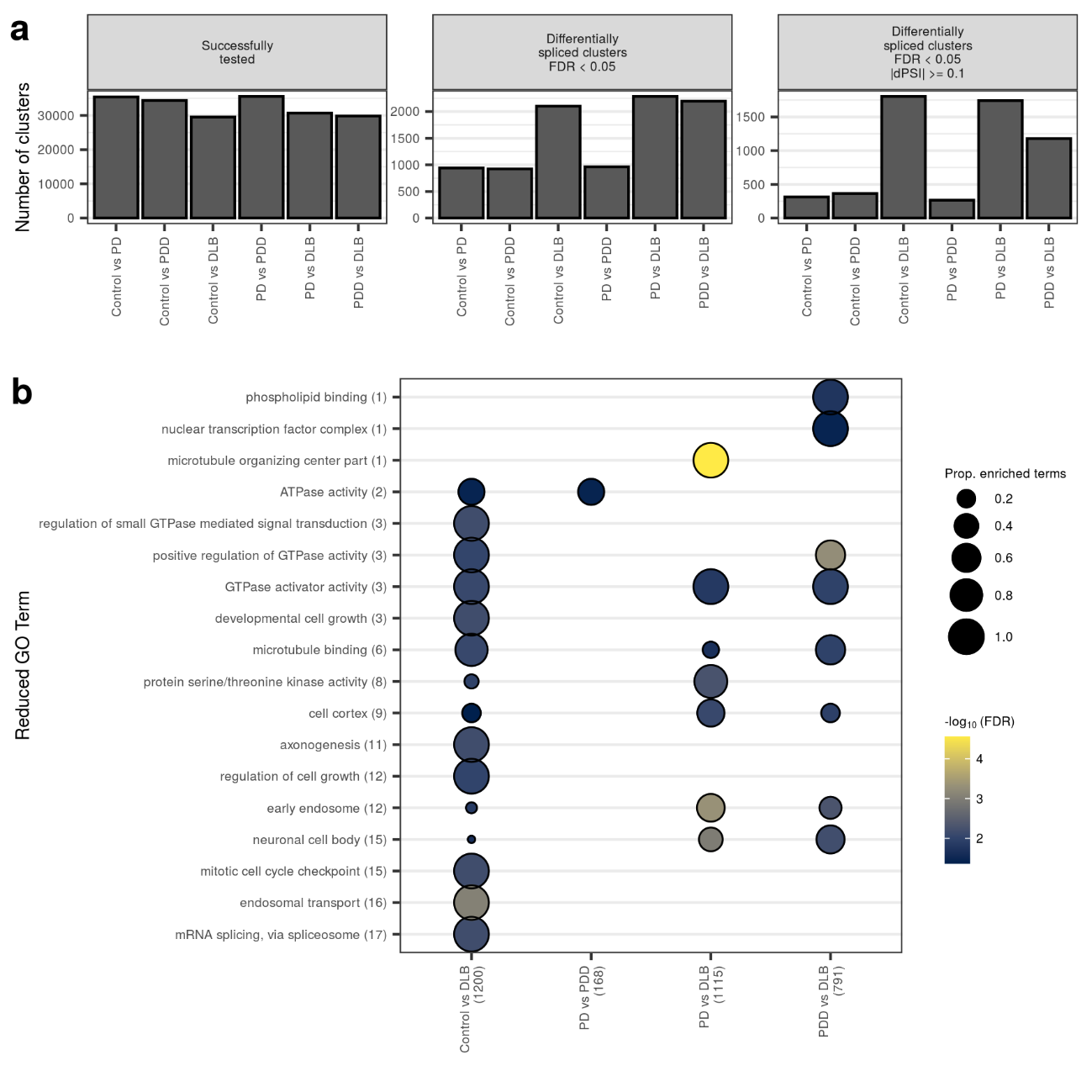


Supplementary Fig. 12 Differential splicing across disease groups.

(a) Number of clusters that were successfully tested and found differentially spliced (DS) following correction for changes in cell-type proportions. (b) Reduced gene ontology (GO) terms associated with genes found DS across pairwise comparisons (FDR < 0.05, |∆PSI| >= 0.1). The number of DS genes is indicated in parentheses on the x-axis. Original GO term enrichments (referred to as “child terms”) were grouped using semantic similarity. The number of enriched child GO terms assigned to each parent term across pairwise comparisons is indicated in parentheses on the y-axis. Size of dot indicates the proportion (prop.) of enriched child terms within a pairwise comparison, which is derived by dividing the number of enriched child terms by the total number of child terms assigned to a parent term. Fill of dot indicates the -log_10_(FDR) of the most significant child term associated with the parent term within that pairwise comparison. Results available in Supplementary Table 11. dPSI, delta (∆) percent spliced in.


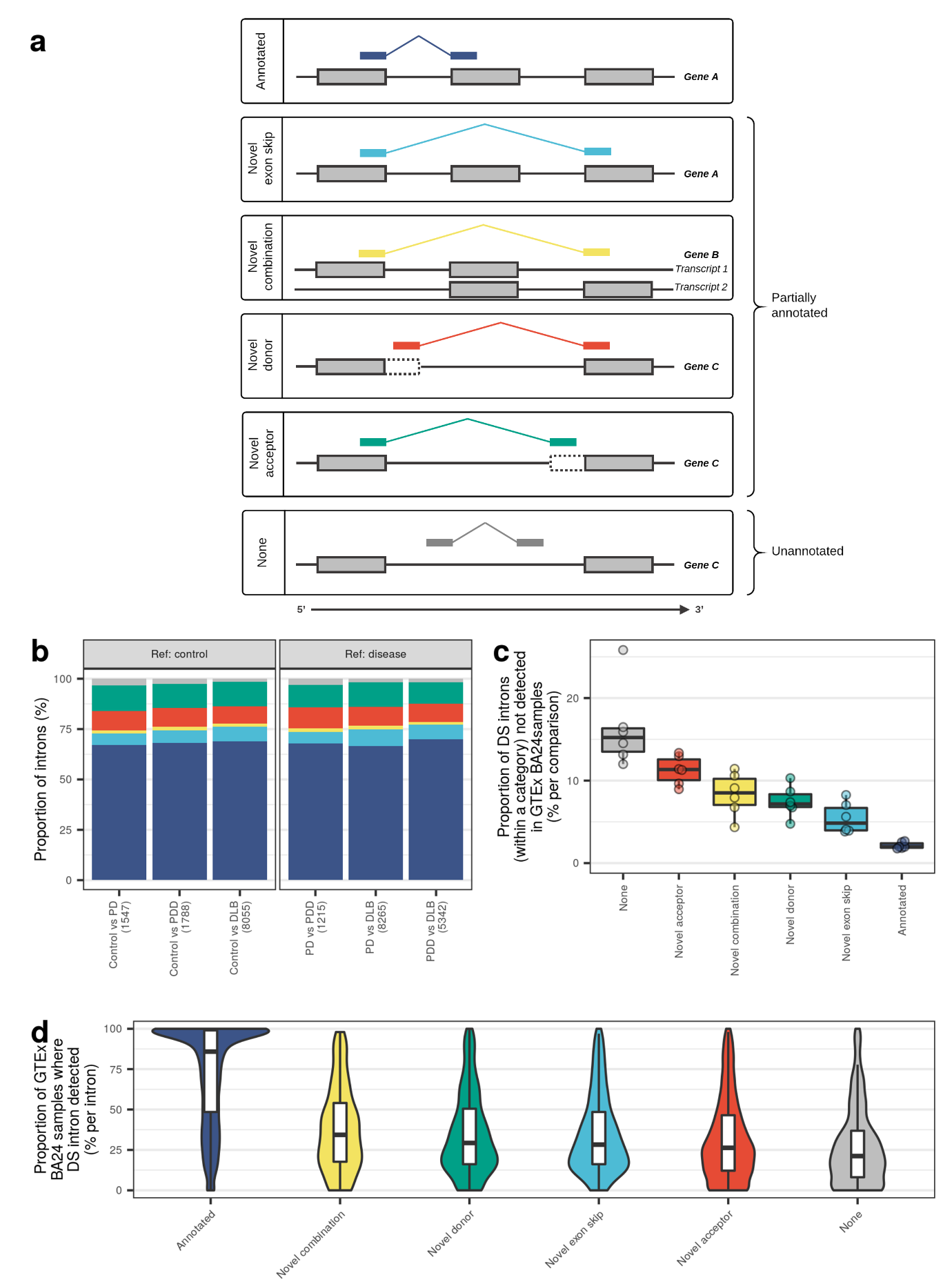


Supplementary Fig. 13 Annotation of differentially spliced introns.

(a) Schematic illustration of the different categories of splicing event. Junction reads used to define Leafcutter introns were annotated based on their relationship to the annotated transcriptome (Ensembl v97). Here, the annotated transcriptome is illustrated by the grey-filled boxes. Annotated junctions have donor and acceptor splice sites that match the boundaries of an existing intron. Likewise, novel exon skip and novel combination junctions have donor and acceptor splice sites that overlap known exon boundaries derived from exons contained within the same transcript, but they represent introns which are not found in the set of annotated introns. They are distinguished by whether or not their donor and acceptor splice sites overlap exons derived from the same transcript. Novel donors and novel acceptors are junctions where only one end (3' or 5', respectively) matches the boundary of a known exon. All novel events are considered partially annotated. Unannotated junctions ("None") have neither end overlapping a known exon. (b) Number of introns assigned to each category of splicing event as a proportion of all introns within the subset of differentially spliced intron clusters (FDR < 0.05, |∆PSI| ≥ 0.1). The total number of junctions in each comparison is indicated in parentheses on the x-axis. (c) Number of differentially spliced (DS) introns that were undetected in a GTEx sample as a proportion of all DS introns within a category of splicing event. (d) Number of GTEx-derived anterior cingulate cortex (BA24) samples where a DS intron was detected as a proportion of the total number of BA24 samples (n = 99). Proportions in (c) and (d) are ordered by median from highest to lowest. ∆PSI, delta percent spliced in.


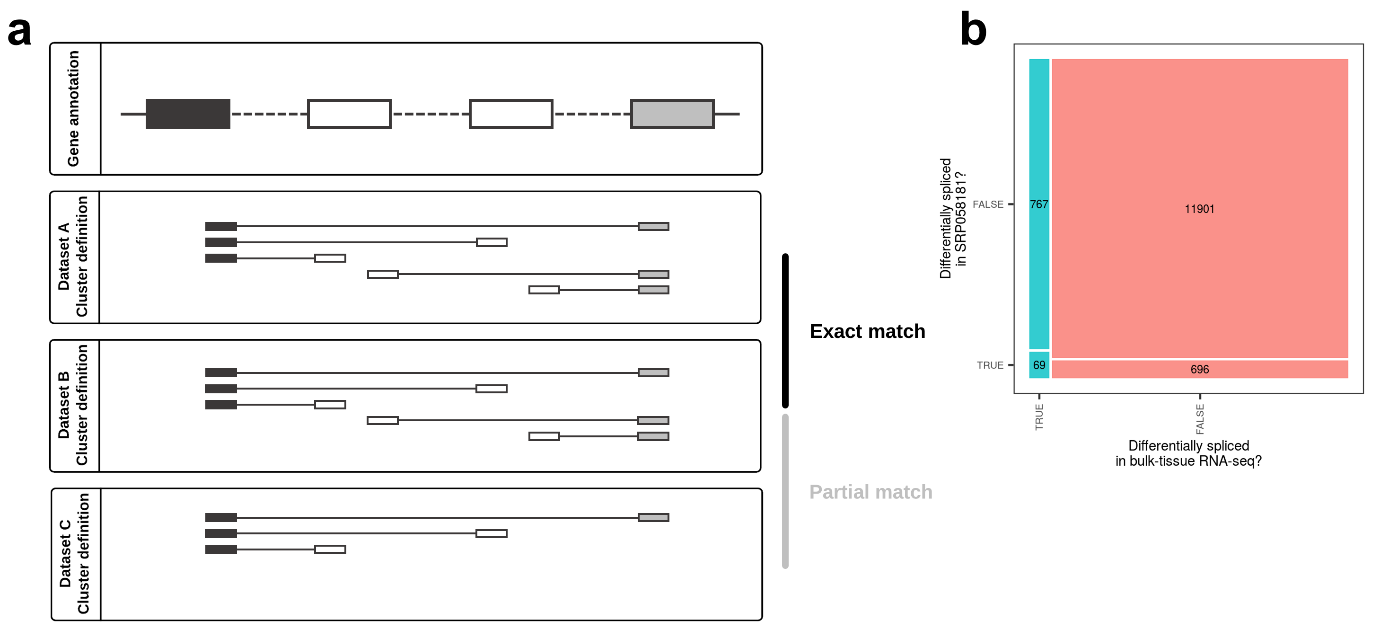


Supplementary Fig. 14 Defining replication across intron clusters and datasets.

(a) Cluster definitions across datasets may vary; thus, comparisons of cluster definitions between datasets can yield exact, partial or no matches (no matches not illustrated). An exact match is defined as an intron cluster that contains the same introns, as determined by their splice donor and acceptor sites, across both datasets. In replication analyses, only exact matches between our dataset and the replication dataset from recount2 (recount ID: SRP058181) were carried forward. (b) Contingency table of differential splicing in our bulk-tissue RNA-sequencing and in the recount2 dataset, SRP058181. Only clusters that matched exactly across the two datasets were used to construct the contingency table. This yielded a total of 13,433 exactly matching intron clusters, 836 of which passed FDR < 0.05 in the discovery dataset. Unadjusted p-values in the replication dataset for these 836 overlapping clusters were then FDR-corrected, and any of the 836 that passed FDR < 0.05 in the replication dataset were considered validated. For gene-level analyses, only those validated intron clusters with ≥ 1 intron that shared the same direction of effect across both datasets were carried forward.


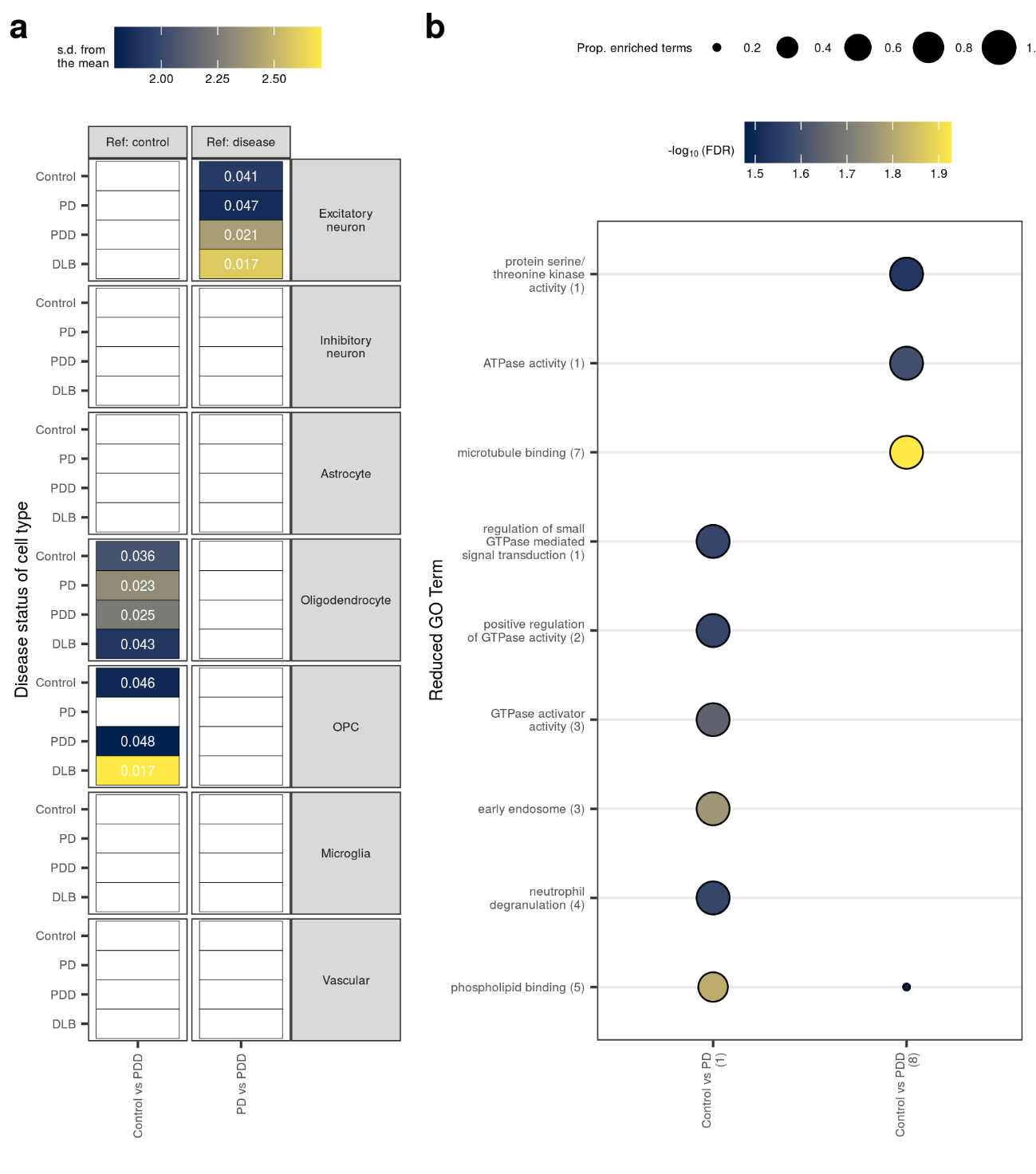


Supplementary Fig. 15 Cell-type and pathway enrichment analyses of validated differentially spliced genes.

(a) Enrichment of validated differentially spliced (DS) genes in cell types derived from each disease group. Enrichments were determined using expression-weighted cell-type enrichment (EWCE). DS at a gene-level was considered validated provided intron clusters matched exactly between our bulk-tissue RNA-sequencing and the SRP058181 dataset, passed FDR < 0.05 in both datasets, and at least one intron in the cluster shared the same direction of effect across both datasets. The x-axis denotes the groups compared in the differential splicing analysis, while the y-axis denotes the cell type and the disease status of specificity matrix from which it is derived. Standard deviations (s.d.) from the mean indicate the distance (in standard deviations) of the target list from the mean of the bootstrapped samples. No results survived FDR correction (FDR < 0.05); displayed are unadjusted p-values. Results with unadjusted p > 0.05 were coloured white. (b) Reduced gene ontology (GO) terms associated with validated DS genes; the number of DS genes is indicated in parentheses on the x-axis. Original GO term enrichments (referred to as “child terms”) were grouped using semantic similarity. The number of enriched child GO terms assigned to each parent term across pairwise comparisons is indicated in parentheses on the y-axis. Size of dot indicates the proportion (prop.) of enriched child terms within a pairwise comparison, which is derived by dividing the number of enriched child terms by the total number of child terms assigned to a parent term. Fill of dot indicates the -log_10_(FDR) of the most significant child term associated with the parent term within that pairwise comparison. Results of EWCE and pathway analyses are available in Supplementary Table 10 and Supplementary Table 11, respectively. OPC, oligodendrocyte precursor cell.


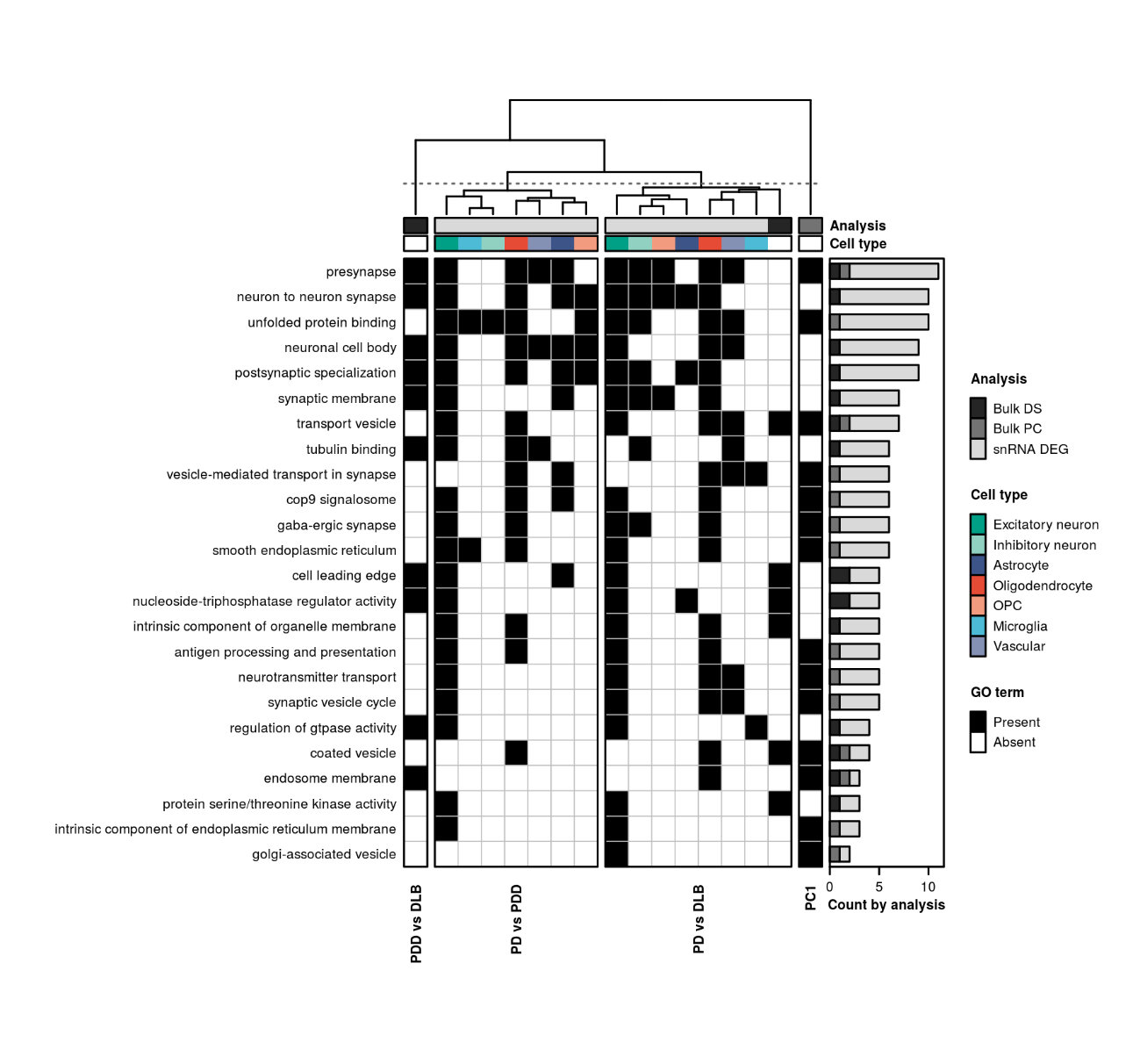


Supplementary Fig. 16 Pathway sharing between disease states.

Clustering of shared pathway enrichments using genes identified across the three main analyses (represented by grey bar entitled, “Analysis”). These included: bulk-tissue differential splicing (“Bulk DS”, Supplementary Fig. 12); gene contributions to bulk-tissue gene expression PC1 (“Bulk PC”, Supplementary Fig. 6); and single-nucleus differential expression (“snRNA DEG”, Fig. 3). Pathways (in rows) from all three analyses were filtered to include only those that appear across more than one type of analysis. Pathways are ordered from highest to lowest by the number of gene sets in which they are enriched (as displayed in the bar plot on the right-hand side). Gene sets (in columns) are clustered using hierarchical clustering on the Pearson correlation between gene sets (pathways were encoded with a binary 1 for “Present” or 0 for “Absent”, represented on the plot by black and white, respectively). Gene sets derived from differential splicing (Bulk DS) were collapsed across our own dataset and the validation dataset, resulting in one gene set (column) per pairwise comparison. Likewise, gene sets derived from up- and down-regulated single-nucleus DE gene sets were collapsed across cell types (represented by the coloured bar entitled, “Cell type”), such that each cell type was represented by a single column. OPC, oligodendrocyte precursor cell.

# Supplementary Note

## Validation of differentially spliced introns in GTEx

### Methods

Junction read counts from 99 GTEx-derived anterior cingulate cortex samples were accessed from recount2 (recount accession ID: SRP012682; GTEx v6) [98,111]. Paired-end 76-bp sequencing was applied to each sample, with a mean depth of 94.5 million read pairs per sample. All samples were of high quality with RIN values ranging from 5.5-8.9 and a median of 6.8. A DS intron was considered “detected in a GTEx sample” if its donor and acceptor splice sites precisely matched that of a junction with a count > 0 in a GTEx sample. Thereafter, two proportions were calculated: (i) the proportion of DS introns not detected in a single GTEx sample and (ii) the proportion of GTEx samples in which a DS intron was detected. The first proportion was calculated separately for each pairwise comparison by dividing the number of undetected DS introns by the total number of DS introns within a category of splicing event. The second proportion was calculated separately for each intron in a pairwise comparison by dividing the number of GTEx samples in which the intron was detected by the total number of GTEx samples.

### Results

To determine whether DS introns were commonly observed in unaffected control tissue, a reference set of 99 control anterior cingulate cortex samples derived from the GTEx project was used. Across comparisons, between 3.8-5.4% of all DS introns went entirely undetected in GTEx samples. Detection rates varied across different categories of splicing event. Depending on the category of splicing event, anything between 1.8-26% of DS introns assigned to the category went entirely undetected in GTEx samples (**Supplementary Fig. 13c**). This proportion was lowest for annotated and highest for unannotated categories, as might be expected under the assumption that an event that does not exist in the reference transcriptome remains unannotated by virtue of the low likelihood of detecting it. Of those DS introns that were detected in GTEx samples, 50% of annotated, partially annotated and unannotated events were observed in greater than 85.9%, 28.3%, and 21.2% of GTEx samples, respectively (**Supplementary Fig. 13d**). Thus, despite the relatively high proportion of partially annotated DS introns, the ability to detect these events in larger control cohorts suggested these were biologically relevant splicing events.

## Replication of differentially spliced introns in GTEx

### Methods

Replication of differential splicing was performed using a replication dataset (see **Processing of PD case-control replication dataset**). Junction read counts were accessed from recount2, filtered to remove any regions that overlap ENCODE blacklist regions [95], and converted to .junc files. Intron clustering (which yielded 37,021 clusters encompassing 128,800 introns) and differential splicing were performed using the same parameters as above. As intron cluster definitions are dataset-dependent, only those intron clusters that matched exactly between the discovery and replication dataset were used for replication purposes. An exact match was defined as an intron cluster that contained the same introns, as determined by their splice donor and acceptor sites, across both datasets. This yielded a total of 13,433 exactly matching intron clusters, 836 of which passed FDR < 0.05 in the discovery dataset. Unadjusted p-values in the replication dataset for these 836 overlapping clusters were then FDR-corrected, and any of the 836 that passed FDR < 0.05 in the replication dataset were considered validated (**Supplementary Fig. 14**). For gene-level analyses, only those validated intron clusters with ≥ 1 intron that shared the same direction of effect across both datasets were carried forward.

### Results

Replication of differential splicing was performed using the same external PD case-control bulk-tissue RNA-sequencing dataset used in replication of deconvolution results. Only those intron clusters that were found to exactly match between datasets were used for replication (n = 13,433 intron clusters). Of these, 836 and 765 were DS (in at least one pairwise comparison) in our dataset and the replication dataset, respectively, with 69 shared between both (p-value = 0.001956; odds ratio = 1.53; 95% CI = 1.17-1.99; Fisher’s exact test; **Supplementary Fig. 14**). We performed cell-type and pathway enrichments on genes containing a shared validated DS intron cluster with at least 1 intron with the same direction of effect in both datasets (*n* unique = 15 genes). Among cell-type enrichment tests, no gene sets passed FDR correction. However, nominally significant enrichments were observed in oligodendrocytes using genes found DS in PDD compared with control, similar to what we observed with our own dataset (**Supplementary Fig. 15a**). Furthermore, several pathway enrichments observed were related to phospholipid binding, endosomes and GTPase activity (**Supplementary Fig. 15b**).
